# GITAD 2020: quality assurance test through 20 years of experience

**DOI:** 10.1007/s00414-022-02802-4

**Published:** 2022-02-22

**Authors:** Juan Carlos Álvarez, Christian Haarkötter, María Saiz, Xiomara Gálvez, María Isabel Medina-Lozano, José Antonio Lorente

**Affiliations:** Laboratory of Genetic Identification, Department of Legal Medicine, Toxicology and Physical Anthropology, Faculty of Medicine, University of Granada, Avd. de la Investigación 11, 18016 - PTS Granada, Spain

**Keywords:** AICEF, Forensic genetics, GITAD, Proficiency testing, Quality control

## Abstract

GITAD (*Grupo Iberoamericano de Trabajo en Análisis de DNA*) was founded in 1998 as the first operational group of AICEF (*Academia Iberoamericana de Criminalística y Estudios Forenses*), formally created in 1999. The mission and the vision of GITAD are to promote the development of forensic genetics in Ibero-American countries and to achieve the maximum level of innovation and quality in each country, and with that aim, a proficiency test was developed. Since 1999, the member laboratories receive four reference samples with the objective of obtaining the genetic profile with their routine protocols, a theoretical exercise since 2003, and since 2007, it was incorporated a forensic sample, which changes every year. The consensus results and the different discrepancies are discussed in an annual meeting. This article illustrates the evolution of the proficiency test through 20 years from different points of view: the increase of participant laboratories, the evolution of the different DNA typing techniques reported by the Ibero-American participant laboratories, the challenges that the proficiency test have met, and future perspectives for a continuous improvement of the proficiency test, especially regarding its accreditation under ISO 17043.

**Supplementary Information:**

The online version contains supplementary material available at 10.1007/s00414-022-02802-4.

## Introduction

The Ibero-American Working Group on DNA Analysis (GITAD, *Grupo Iberoamericano de Trabajo en Análisis de DNA*) was founded during a scientific meeting held in Orlando, Florida in October 1998 by 11 representatives from various countries including Chile, Argentina, Uruguay, Brazil, Venezuela, Colombia, Costa Rica, El Salvador, Mexico, Puerto Rico, and Spain [1], with currently 31 Ibero-American public institutions members that are already members of AICEF (*Academia Iberoamericana de Criminalística y Estudios Forenses)* from different countries of Ibero-America. The mission of GITAD is to promote the development of forensic genetics in Ibero-American countries, with a special consideration of the differing laws of each country and with strong compliance of quality standards. The vision of GITAD is for every member country in the coming years to achieve the maximum level of development, innovation and quality in their processes, with benefits for each country’s judicial system. To this end, a recently published study was conducted by the GITAD Database Commission on DNA databases and their regulation in 15 Latin American countries [2].

The GITAD objectives include collaboration between its members (via annual meetings, workshops, exchange of information, etc.) in human genetic identification, standardisation of analytical techniques, capacitation and promoting the exchange of experiences, and annual proficiency testing. These networks need to be developed to ensure the application of forensic genetics at the highest scientific and qualified level in Ibero-America [3]. The annual GITAD proficiency test is free and has been undergoing developing since 1999. The test is internationally recognised, and the number of participating laboratories has increased every year. In the wake of the international symposium in 2006 between AICEF and the Indo-Pacific Association of Law, Medicine and Science, this proficiency test was introduced to Asia, and numerous laboratories and countries have subsequently participated, including India, China, Indonesia, The Philippines, South Korea, Sri Lanka, Nepal, Taiwan, Malaysia, and Australia. Furthermore, AICEF is part of the International Forensic Strategic Alliance [4] (https://www.ifsa-forensics.org/). In this way, participant laboratories are making commitments to be part of this collaborative network, volunteering in order to improve their procedures and results.

Quality assurance, proficiency testing, and troubleshooting are reliable ways to assist laboratories to achieve trustworthy results. A proficiency test is an assessment of the performance of a laboratory analyst, tests that are performed periodically by a DNA typing laboratory. Previously analysed biological samples are submitted to the different laboratories to be tested, with the purpose of evaluating the laboratory’s ability to obtain a result concordant to the previously determined. A proficiency test is internal if it is provided by someone else in the laboratory and external if it is undertaken by an external organisation. The final aim is to test the performance of a laboratory running a set of unknown samples to the analyst but known for the supplier, so a successful completion may guarantee a certain degree of confidence with a real forensic analysis, besides certain challenges and costs are inevitably associated [5]. The basic principles of a blind trial system is the same as a system of quality control, trying to evaluate several problem areas: the ability of an analytical method to give a result and the specificity of the method given the accuracy, precision, and limits of detection of the method [6]. There are four different models of blind proficiency testing [5]: blind/law enforcement, blind/conduit lab, blind analyst, and random audit/reanalysis, each one with its advantages and disadvantages. GITAD Proficiency Test works as a blind proficiency test (participant laboratory system performance is tested).

Proficiency testing is quite a useful strategy to implement in Forensic DNA laboratories, since it promotes accuracy, indicates if the results obtained by the laboratory are correct or not, and helps finding strategies in order to improve the laboratory procedures [7, 8]. There are several national and international organisations that ensure the results of forensic DNA typing laboratories, including GHEP-International Society for Forensic Genetics [9], German DNA Profiling (GEDNAP) [10], and ENFSI [11]. Furthermore, the National Institute of Standards and Technology (NIST), that prepares standards for traceability and calibration, is a valuable tool for internal laboratory controls [12, 13]. Proficiency tests are also essential for programs based on DNA typing [14].

## GITAD proficiency test

The Laboratory of Genetic Identification of the Faculty of Medicine of the University of Granada is responsible for preparing the GITAD proficiency test. All participating laboratories in this laboratory are registered. The laboratory prepares two groups of samples: an obligatory group (M1–M4) and an optional group, the “forensic samples” (M5). The M1–M5 samples are prepared and distributed to each of the participating laboratories. The proficiency test participation is completely free of charge; participants do not have to pay a registration fee or to receive the samples. However, reagents, kits, and assistance to annual meetings are financed by each participant.

The rules of participation are as follows. Every year, there is a formal announcement via email addressed to all GITAD members and other public institution-associated members that previously stated the intent to participate. This email includes a form which the members who are interested on participating in the proficiency test have to fill out, including name of the responsible person, institution, address, and whether they would like to participate in the theoretical exercise and/or the forensic sample. The answer with the filled form has to be sent prior to the end of a previously established deadline.

After that, the proficiency test samples are prepared according to the number of participant laboratories. Four different blood samples are obtained from volunteers of the laboratory personnel, whose DNA is already typed. These samples may be family related or not; however, a certain kinship is aimed to be obtained among samples so different hypothesis and calculations could be performed. The forensic sample (M5) changes every year since it is supposed to be a forensic challenge, and it is decided after a meeting of the organisation staff members. Prior to send the samples, they are analysed by the laboratory with its routine DNA typing techniques. The obligatory stage of the proficiency test consists on analysing samples M1-M4 with the routine techniques of the laboratory and obtaining the genetic profile of them. Optionally, participant laboratories are asked to investigate any kin relationship among samples, so they can eventually formulate the applicable hypotheses and perform the right statistics calculations.

Prepared samples are sent to the different participant laboratories via registered mail in a paper envelope containing samples M1–M4 in a Human ID Bloodstain Card (Whatman™) card (in earlier exercises FTA® (Find The Agent) or filter paper) and M5 if the laboratory signed it in the participation form, individually packaged. A letter with technical instructions is also sent, containing the dispatched material (including a description of the obligatory samples and the forensic sample), and the exercise proposal. As a mandatory part, the participant laboratories have to obtain the genetic profile for samples M1–M4 with their routine DNA markers, describing the extraction, quantification, and amplification methods, and they are also asked to optionally send a copy of the obtained electropherograms in order to assess the cause of any discrepancy. There is no minimal number of DNA markers to be analysed, and the results may be given with their respective statistical calculation (this is optional). In an optional stage, laboratories are asked to establish the possible relationships between M1 and M4, to analyse Y-chromosome and X-chromosome STR markers as well as mitochondrial DNA, to analyse M5 and to establish the possible relationships with the obligatory samples, and to perform a theoretical exercise in an attached Excel file. For result normalisation, allelic frequencies for the most common DNA markers are already given in the exercise. A result form is provided to the laboratories via email, asking the same information to the participation form (laboratory responsible person, institution, address, and optional analyses performed), but also including a form with the used methods (preliminary tests, DNA extraction, DNA quantitation, DNA amplification and DNA detection) and the theoretical exercise.

Once the laboratories have tested the samples and returned their results, they are compared to the previously obtained results and among them so consensus results are obtained by the majority criteria. Optional approaches such as mitochondrial DNA analysis or X-STRs typing are only taken into account if a minimum number of three laboratories are reached (and consensus results are obtained). An annual meeting is held to compare the results and discuss the observed discrepancies, and after that, proficiency test certifications are sent to the participating laboratories with the number of successfully analysed markers (compared with the consensus results). If it happens that participant laboratory results show discrepancies, the certification contains the successful markers, and recommendations according to the observed discrepancies and their nature are made so decisions regarding the obtained results can be taken by the discrepant laboratories. Discrepant markers are also specified in the report with their respective error cause if it is founded by the observation of the electropherograms.

### Samples preparation

The prepared samples are the same for both Latin-America and Asia exercises and they are prepared during the same during the same workday. Samples M1–M4 are placed on filter paper or Whatman™ cards (either Human ID Bloodstain of FTA®) from blood obtained via vein puncture, obtained from volunteers from the laboratory staff, resident students, studies conducted in other research departments or closed cases, or other donors previous informed consent. Since 2007, when it was established in Spain, a procedure for informed consent in biological research, samples are only taken from laboratory staff.

The main challenge is to guarantee the homogeneity of samples. In order to achieve a certain degree of similarity, samples M1–M4 (blood on cards) are prepared by adding 15 µl of blood (which tube is previously agitated during 30 s in a vortex) to the centre of each circle of the card, agitating again during 10–15 s every 10 samples. The homogeneity of sample M5 depends on its own nature so there is no established protocol for it; however, it is well defined when it is established. It is obtained the same as M1–M4.

All reference and forensic samples are tested by the laboratory prior to be sent to the participant laboratories. Testing include routine STR markers, X and Y STRs, and mitochondrial DNA following the validated laboratory testing procedures in order to assess the expected results for the majority of markers that are going to be reported; thus, the obtained results are not part of any posterior consensus. If any reported result is unknown since different DNA markers or kits are employed, in order to obtain a consensus result, at least a minimum of 5 laboratories reporting it and no less than 70% results concordance is required.

### Obligatory exercise

The first problem that participating laboratories have to solve during the proficiency test is determining the samples’ genetic profile. The obligatory samples (four samples with known origin placed on filter paper or Human ID Bloodstain Card (Whatman™) cards) are usually blood stains. To pass the proficiency test, the participating laboratories have to determine the profiles of the four samples for the markers that might be routinely analysed in their laboratories. Furthermore, a description of the method employed in the DNA extraction, quantification, and amplification is required, with the aim of checking the employed techniques and determining the workflow from the moment the sample is received to when the results are submitted. An electronic copy of the resulting electropherograms is also required.

### Forensic sample exercise

In the optional control stage, the laboratories are asked to study the maternal and paternal relationship between the samples by applying at least the HV1 and HV2 regions of the mitochondrial DNA analysis and Y-chromosome short tandem repeats (STRs). Since 2007, a forensic sample has been included in the test (Supplementary material Table 1), consisting of various biological fluids fixed on a variety of surfaces. The forensic samples vary every year: in some cases, an equilibrated mixture of two types of blood; in others, a mixture of semen and saliva and even bone powder. Homogeneity is an important goal in M5, so special considerations according to its own nature are taken in order to guarantee it. For example, 2020 M5 (cigarette butt with male saliva) was prepared soaking the smoked cigarette butt ends in 50 ml of male saliva during one second in agitation. Informed consent considerations are the same as in samples M1–M4, already explained.

### Theoretical exercise

Since 2003, the last stage of the test provides a theoretical problem for sample interpretation and statistical treatment (see Supplementary Material Table 2). Initially, the theoretical exercise was related to the observed relationship among M1–M5 samples but later different genetic profiles were provided with the exercise statement. In some cases, the problem involved different kinship exercises (paternity, siblings, etc.), while others involved sexual assault. The individuals’ genetic profile, study sample, and population genetic database are provided.

## Proficiency test evolution

### Increase in the number of participating laboratories

**Fig. 1:**
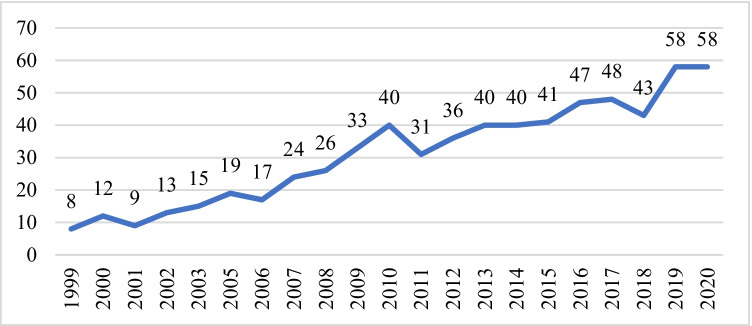
Evolution of the number of participants since GITAD foundation

For the first proficiency test in 1999, there were eight participating Ibero-American laboratories. Over the past two decades, there has a progressive increase in registered participants, reaching 58 in 2019 and 2020 (Fig. 1). It has to be noted that there are more participant laboratories than GITAD members since each laboratory may participate with more than one analyst.

Most of the participating laboratories in recent years have been from Mexico (12%), Spain (14%), and Brazil (56%), the latter of which recognises the GITAD proficiency test as an official proficiency test for state laboratories. Other participating countries include Peru, Colombia, and Argentina (2–5%). A parallel proficiency test, with the same group of reference and forensic samples, is performed in Asia (e.g., India, Taiwan, Indonesia, Thailand, and China).

### Evolution of the analytical techniques

This section covers the evolution in the preliminary tests, DNA extraction, quantification, and amplification techniques, result visualisation, genotyping software, calculation software, and discrepancies employed by the laboratories (data not shown). This information has been extracted from the reports of the participant laboratories from 1999 to 2013, when a form covering these aspects were introduced. It has to be noted that the exercise was not conducted in 2004. As an illustration of nowadays techniques employed in Ibero-America, see Supplementary material Figs. 2–4 (data is presented as the percentage of reports from the participant laboratories including a certain technique or protocol). Supplementary material Figs. 5–12 show the evolution of the different techniques from 1999 to 2020 (there is no data available for the first years).

For preliminary tests*,* human blood-specific methods are the most frequently used, followed by prostate-specific antigen detection. The organic extraction protocol with phenol, chloroform, and isoamyl alcohol was the most commonly reported protocol from 2003 to 2013, when it was replaced by automate extraction platforms. Quantifiler™ Trio is the preferred quantification options since 2015; however, being PowerPlex™ Fusion the most common STR amplification approach, the same as PowerPlex® Y23 for Y-STRs. The use of X-STRs has been scarcely reported, with only one laboratory in 2016 and five in 2019. Mitochondrial DNA analysis has also been scarcely reported, with seven laboratories in 2018.

Next generation sequencing platforms were reported by only two of the participant laboratories, being MiSeq™ FGx (Illumina, San Diego, CA, USA) in 2015 and Ion S5™ System (Thermo Fisher, Waltham, MA, USA) in 2019 the informed platforms. Until now, no NGS platform has been reported in Asia. Accessibility issues may be proposed here: participants are public justice-related institutions more than research facilities, so they may be involved in specific economic procedures plus certain agreements between the administration authority and reagent providers may be applied. In addition, GITAD proficiency test expects the routine techniques to be employed and NGS data is a relatively new incorporation to forensic casework. However, more NGS employment is expected in the near future.

Not only technology has evolved, but also the proficiency test itself: sample nature, preparation and quantity (see in Supplementary material Table 1 the decreasing volume of sample), sample selection criteria, and reports processing data are some aspects of that evolution.

### Laboratories performance

**Fig. 2:**
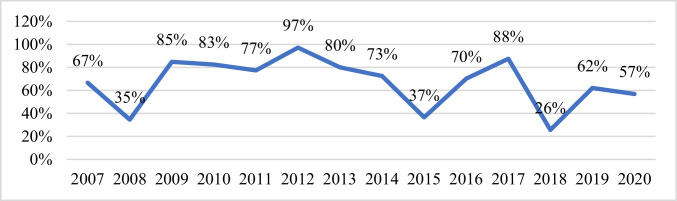
Percentage of participant laboratories analysing the M5 sample

Several aspects can be studied here, such us how many laboratories undertake the forensic sample, and how many discrepancies has been observed in the exercises, as well as the nature of those discrepancies. Firstly, there has been an increasing tendency in the number of laboratories performing the forensic sample analysis (M5), with a maximum of 42 laboratories in 2017 (mother-and-son mixture) (Fig. 2). In terms of the percentage of laboratories analysing the forensic sample, more than half of the laboratories undertake this optional stage of the quality sample.

**Fig. 3:**
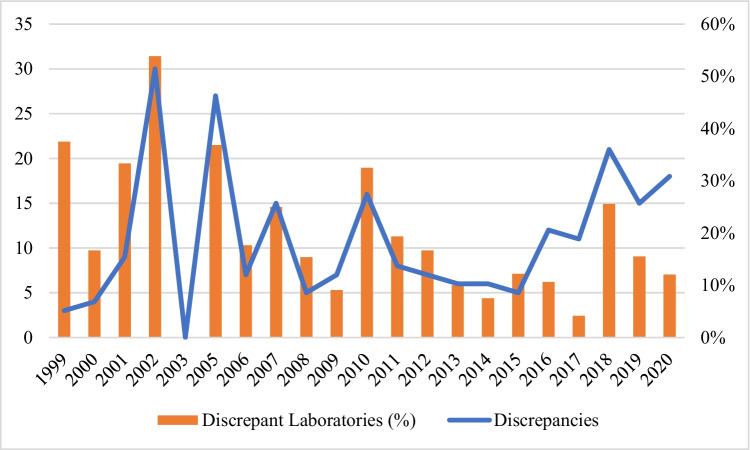
Number of discrepancies and percentage of laboratories reporting discrepant results in M1–M4 by year

Secondly, there has been a slightly increasing tendency in the number of reported discrepancies (Fig. 3). Curiously, however, the number of laboratories reporting discrepant results tends to be lower every year. The growing number of discrepancies could be explained by the larger number of participating laboratories; the more laboratories undertake the proficiency test, the more discrepancies are observed. Nevertheless, this observation does not translate into a lack of security in the various laboratories, given that the number of laboratories reporting discrepant results is lower every year. Thus, Fig. 3 could be divided into two separate phases. During the first phase (2001–2010), characterised graphically by a sawtooth wave, discrepancies were present and were discussed in the annual meetings during which solutions were proposed, resulting in the following year’s proficiency test showing a significant reduction in discrepancies. The second phase covers the last years of the proficiency test, with a slight increase in discrepancies and a reduction in the number of laboratories reporting discrepant results, given that discrepancies were concentrated in a few laboratories. A slight positive correlation was found between the number of discrepancies and the number of discrepant laboratories (*p*-value > 0,001 for a 95% confidence interval, r = 0.670, calculated with IBM SPSS Statistics 26). Therefore, it seems that the number of errors increases with the number of participants, so errors are not concentrated in a few laboratories. An M5 number of discrepancies are not shown since they are expected to be more numerous given the challenging nature of the exercise; however, the cause of the discrepancies will be discussed next.

**Fig. 4:**
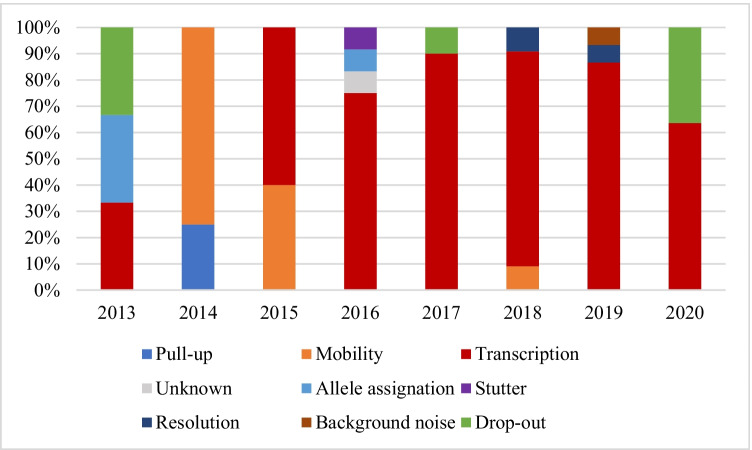
Cause of discrepancies by year (M1–M4). The results are shown as relative percentage of each discrepancy compared to all of them

**Fig. 5:**
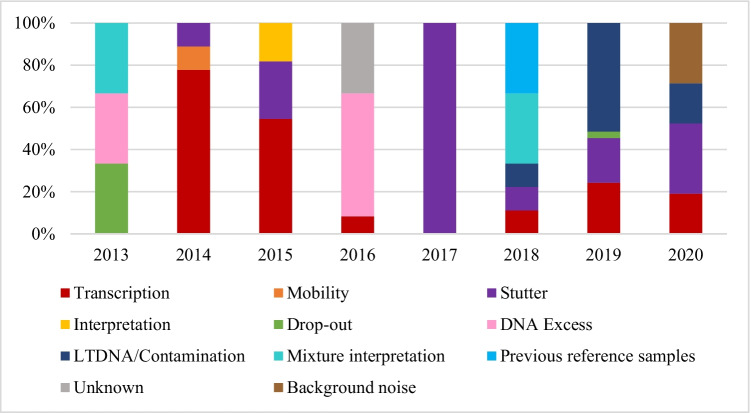
Cause of discrepancies by year (M5). The results are shown as relative percentage of each discrepancy compared to all of them

The various discrepancies observed are shown in Figs. 4 and 5 and explained in Table 1. For comparative reasons, only the last years’ exercises are shown. There were differences between samples M1–M4 and M5; thus, the most common error with the first group of samples was the transcription of the obtained results to the proficiency test form (an error that may be explained by the change of the routine report to the proficiency test one), whereas the forensic sample M5 was the result of the nature of the sample. The most common discrepancies therefore varied over the years, from transcription (2014, 2015) and DNA excess (2016) to mixture, contamination, and stutters during the last 3 years.

**Table 1:** Type of errors found in the exercises

Error type	Description and cause
*Allele assignation*	Alleles contained in the questioned samples and in the allelic ladder runs in a different manner [15]. Genotyping errors are caused by scoring mistakes, biochemical anomalies, degraded DNA samples or contaminated multiplex assays [16]
*Background noise*	A signal not associated with amplified DNA caused by the condition of the polymer and capillary used or by dirt in the capillary window or pump block. Any measurement with a light-detecting instrument is subject of background noise, caused by age and condition of polymer and capillary or dirty capillary windows or pump blocks [17]
*Contamination*	Alleles or entire profiles belonging to staff members, other samples, or external DNA [18]. Many processes may lead to a contamination of genetic profiles (before, during or after the crime event), and they act by mechanisms such as direct contact, aerosols or secondary transfer [19]
*DNA Excess*	Excessive DNA template during PCR amplification that translates into locus imbalance, split peaks, off-scale signal, pull-up peaks, or disproportionate stutters [20]
*Drop-out*	Missing alleles at one or more allelic loci due to low DNA input [21] due to stochastic variation during amplification with low DNA template [22]
*Interpretation*	The result is correct in the electropherogram but has been incorrectly interpreted by the analyst
*Low Template DNA (LTDNA)*	Background DNA unrelated to the sample but still detectable in the genetic analyses [23]
*Previous reference samples*	If the forensic sample is a mixture composed of the reference samples, an error in their analyses will lead to an error in the forensic sample performance
*Mixture interpretation*	Two or more individuals have contributed to the sample, which is detected by the presence of more than two alleles in a locus, extreme peak imbalance, and amelogenin imbalance [24]
*Mobility*	Allelic designation errors due to variations in the travelling DNA molecules through the capillary due to temperature and electrophoresis changes [24]
*Pull-up*	Fluorescence bleeds through into adjacent colour channels due to amplified product excess or insufficient spectral calibration [24]
*Resolution*	Poor shape peaks caused by problems in polymer freshness or the presence of bubbles [24]
*Stutter*	Minor amplification products produced by DNA polymerase slippage[25]
*Transcription*	The laboratory has correctly obtained the expected result; however, it failed to transcribe it properly into the form due to a human error
*Unknown*	Error of an unknown nature or explanation

Finally, between 21 and 70% of laboratories perform the theoretical exercise (from the participant laboratories). The period between 2009 and 2015 concentrates the biggest number of laboratories that participate in the exercise, whereas 2016 is the year with less laboratories undertaking the theoretical case.

## Discussion

### GITAD proficiency test recognition

The Y Chromosome Haplotype Reference Database (YHRD), whose goals are to generate a reliable Y-STR haplotype frequency database, assess the worldwide male population stratification, and provide tools for research in Y-STRs and Y-SNPs, has established on its webpage two preconditions for submitting an haplotype: (1) a laboratory accreditation certificate and (2) having passed a proficiency test that includes YSTR analysis, with GITAD one of the accepted proficiency tests [26]. Furthermore, participating in and succeeding with the GITAD proficiency test is a prerequisite for adding a genetic profile to the Brazil Federal Police genetic database (*Rede Integrada de Bancos de Perfis Genéticos*, RIBPG) [3].

### Number of participating laboratories

The number of participating laboratories has been constantly growing since the implementation of the proficiency test in 1999, when there were 8 participating laboratories. This number was doubled in 2005 and again in 2010 to 40 participating laboratories, a number that has remained fairly steady in the following 5 years, reaching a total of 58 laboratories in 2019 and 2020, the highest number in the proficiency test’s history. The growing number of applicant laboratories suggests their interest and satisfaction with the proficiency test.

### DNA typing evolution

One of the aims of this article was to call attention to the evolution of the various DNA typing techniques employed in Ibero-America, which is very close to the evolution of forensic DNA analysis. Despite the lower use of preliminary tests, there was a tendency to use a wider range of techniques for various biological substances (blood, saliva, and semen). Although the use of a preliminary test depends on the forensic sample, human blood preliminary tests are the most common among the various exercises, especially the Hexagon OBTI kit and Adler and Teichmann methods. Although less frequently, semen and saliva detection are still employed. Surprisingly, quite obsolete methods such as the Adler (which uses a benzidine reaction with blood in the presence of hydrogen peroxide) and Teichmann tests (hemin crystals observed through microscopy) are commonly used. However, the number of participating laboratories using any presumptive test is still low, or at least it has not been reported. Presumptive tests have a major role in forensic sciences in general and in forensic genetics in particular, because the association between a DNA profile and a body fluid is not fully associated, even if a preliminary test yields a positive result. The augmented sensitivity of current DNA typing laboratories makes it possible to obtain a touch DNA profile previously deposited by a body fluid stain at the crime scene; therefore, analysts need to consider all DNA and non-DNA evidence [27]. An example of this is 2016 M5, a non-human bloodstain in a human touch DNA background, topic that will be covered later.

DNA extraction has evolved from manual, time-intensive, and laborious protocols to automated DNA extraction and commercial kits, aimed at fast, reliable, and efficient solutions for optimal extraction. DNA extraction is the most important step in DNA typing because it has the greatest impact on the ultimate goal of obtaining a good DNA profile, which is possible only by obtaining the largest quantity and highest quality of DNA. Classical DNA extraction methods such as Chelex® and phenol–chloroform are still employed by forensic scientists for certain samples; however, manual and automated DNA extraction kits (developed in the early 2000s) are the most commonly used. DNA extraction from forensic samples, unlike clinical samples, is not completely automated or standardised, since forensic samples differ in the type of body fluid, nature of the tissue, substrate, nature of the crime, quantity of biological material, and source. The chosen method should therefore yield a high amount of DNA, removing or reducing PCR inhibitors that may have an impact on downstream analyses [28].

Many of the laboratories reported no DNA quantification step given that automated systems with a sample normalisation step or FTA® purification reagents are used. The use of various commercial kits has been reported, but there has been a tendency to use TaqMan® chemistry and kits that offer a variety of targets. The main usefulness of DNA quantification with casework samples is the optimisation of DNA input in the various STR amplification kits, thereby providing information on the quantity and quality of samples.

DNA amplification is probably the most variable stage in the DNA typing process, with several strategies available, each with a variety of commercial kits. However, there is a clear tendency to use more markers, and Y-STRs analysis has become a routine technique, despite mitochondrial DNA analysis; X-STRs studies have been less often reported. In addition to the aim of using more strategies, we should note the introduction of next-generation sequencing in the laboratories, given that it might become a common approach in the future.

DNA visualisation has evolved with the various available systems, although there is a clear preference for more fluorochromes (which translates into more markers in one run), more flexibility (the required maintenance for genetic analysers is increasingly flexible), and more capillary tubes so that more samples can be analysed. The use of data analysis software, however variable, shows a clear tendency towards specific forensic applications. In contrast, freeware and open-source options are preferred for statistics software.

Concordance problems among the various commercial kits are another subject of discussion. NIST and several researches have conducted concordance testing to establish the presence of concordance problems among the various available kits, obtaining percentages > 99.0%. The observed problems are normally reported to the commercial vendors so that they can improve their kits [29].

The most widely used statistics applications (in order to give a probability to the result given by the laboratories) were PATCAN (from 2003 to 2011) and Familias (from 2012 to the present). Statistic software is used mostly in order to solve the theoretical exercise, yet some of the laboratories give the obligatory and forensic exercise results with a probability or LR obtained with those programs if any biological relationship was observed among samples. Finally, 83% of the participant laboratories reported the use of freeware software, while 14% of them reported the use of commercial software (the rest reported calculations performed by hand).

### GITAD Forensic exercises

Several of the exercises with forensic samples during the past 14 years they were conducted deserve a mention. The human bone fragment and bone powder samples (2007 and 2008 M5) that were sent the first years were very well evaluated by the participating laboratories. They were interesting samples because they probed the laboratories’ capacity for dealing with challenging samples; however, these samples are significantly more difficult to send than regular samples due to customs regulation and prohibitions; in addition, homogeneity is more challenging to achieve with the growing number of participant laboratories. Nevertheless, it was an appreciated matrix and its re-implementation is nowadays under evaluation by the proficiency test organisation staff.

The mixture exercises were the most common forensic sample, many of which were composed of samples from genetically related individuals, which made the analyses and interpretation even more complex. This type of experience helps the laboratories to gain awareness to the inherent difficulties in the analysis and interpretation of mixtures. When comparing results, this experience helps evaluate and compare strategies so that a more successful mixture interpretation could be achieved. In 2013 M5 (bloodstain mixture of M4 and M4 subject’s daughter), for instance, many laboratories detected the mixture completely, some of them detected the alleles with some drop-out or drop-in, and a few laboratories did not report any mixture. In 2014 (bloodstain mixture of M4 and M4 subject’ sister) and 2015 (M4 and his/her brother), transcription and stutters were the most common errors observed; however, it was difficult to assess given that many of the laboratories did not send the electropherograms, an issue that needed to be corrected. For 2018 M5 (woman hair soaked in M4 blood) preliminary tests, it was expected positive in human blood and negative for any other fluid, the identification of two different genetic profiles (M4 in hair wash and MX in hair) and no family relationship between M4 and MX. However, four types of results were reported: the same as expected (type 1), mixture profiles in different proportions (type 2), the obtention of only M4 genetic profile (type 3), and no results or contamination results (type 4). In the end, conditional transposition in LR calculation was observed, different hypotheses were proposed, and divergent results and conclusions were achieved.

The forensic sample with pork blood in a human touch DNA background (2016 M5), as well as its discussion, exposed the participating laboratories to the challenges and issues regarding DNA transferences and the importance of performing preliminary detection and orientation tests on biological samples, particularly for determining whether or not the sample has a human source. In this exercise presumptive tests, positive results for blood (not specific) but negative results for human blood or any other human fluid were expected (here lies the importance of immunochromatographic tests for screening samples given their specificity for detecting human blood), and it was also expected not only the obtention of only one genetic profile as background DNA, but also the identification of *Sus scofra* species by mitochondrial DNA analysis, so no association between genetic profile and body fluid was anticipated. The challenge of this forensic sample was precisely the dangerousness of associating a genetic profile to a certain body fluid or origin, an issue that has already being evaluated [30]. The reported results could be classified in three groups: negative, partial profile and complete profile, and the laboratories who reported the performance of a preliminary test noticed that there was blood, however not human. A common error in this exercise was DNA excess in amplification, linked to the fact that DNA quantification was poorly reported.

### Theoretical exercise

Theoretical exercises have become a very much appreciated instance in proficiency test programs of different scientific groups, since they allow to detect statistical problems and software errors in routine reports of forensic genetics laboratories [31]. Since the theoretical exercise was implemented, almost half of the participant laboratories perform it, with varying results. In general, three are the main committed mistakes: conditional transposition, hypothesis considered, and conclusions. Conditional transposition is also called “prosecutors’ fallacy”, well known among forensic scientists [32], an issue that should be avoided in laboratory reports.

Thus, there have been interesting conclusions to these exercises since its implementation, for example, 2013 theoretical exercise (half siblings) including an incompatibility in one single DNA marker, a challenge that laboratories assumed in three different ways: eliminating the marker from calculations, including it with its correspondent mutation rate, or giving results without any explanation.

In the mixtures of theoretical cases (2014), the electropherogram information (peaks and RFU height) is given as well as the genetic profiles and the population genetic database. Questions about the number and the genetic profiles of contributors and the difference between stutters and alleles are asked. Besides, there is a huge variability in answers: there was a wide range of answers when giving the proportion of the mixture and a huge variability when deducing the genotypes from electropherograms. However, the majority of the laboratories noticed correctly the mixture and present alleles.

Other exercises such as 2015 theoretical case (putative father and son in a possible incest case) offer an extra challenge to participant laboratories. In this case in particular, no mother genetic profile was given, so participants were forced to use different statistical calculations to the routine ones, and once again, a huge variability was observed in reports. The majority of the participant laboratories did not use any special formula, however being warn of the possibility of an incest case. The most common error was, once again, conditional transposition.

Finally, in 2016 exercise (sisterhood), the most common error was an inadequate hypothesis contrast, since not all the possibilities were taken into account. Furthermore, conditional transposition error was widely distributed among answers, and the same can be said about 2018 exercise (grandparenthood). Disaster Victim Identification (DVI) theoretical exercise in 2019, however being correctly performed by almost every participant laboratory, showed a wide range of results. The different family profiles that were given in the exercise were close family members: father and mother, spouse, daughter/son, or siblings.

The evolution of the theoretical exercise performance has stated the importance of statistics, calculations, hypotheses formulation, and conclusions formulation in forensic genetics, so those topics has been emphasised in several GITAD annual meetings by discussing them in a general assembly or by offering specific workshops related to these issues.

### Challenges sending the samples

There have been several problems in sending the samples to the participating laboratories. First, biological samples are usually classified as dangerous goods by most countries and are therefore particularly treated by the customs officials of the various receiving countries. Second, custom offices present a common problem given that shipments are often returned from foreign countries. This problem was magnified during the last exercise due to COVID-19 restrictions for receiving packages from foreign countries, with most of the letters subject of quarantine for several days before delivery.

### Participant laboratories improvement

GITAD exercise is not just about participating in a proficiency test: collaboration and improvement are two of its main pillars. Indeed, participant laboratories may have changed in one or more of the following aspects.

Firstly, laboratories have gained in partnership: being part of a forensic genetics network with colleges from their surroundings countries, attending meetings that stress on the latest advancements or issues, workshops, information exchange between colleges, or protocol unification are some examples of this kind of collaboration.

Secondly, discrepancies may be useful to participants since they may reveal a critical point in their protocols or procedures, so they are able to change them in order to improve them. Participants may also discover malfunctions in their instruments and equipment, for example, a participant realised they had electrophoretic mobility problems or lack of resolution for distinguish allelic variants with their proficiency test results. Other laboratories may change their protocols when they notice an error, for instance, when participants observed transcription errors as one of the main cause of discrepancies, they changed their protocols so now results are peer-reviewed before being reported. In addition, as many laboratories are already accredited under ISO 17025, a formal discrepancy protocol may be activated, so it promotes revisions, actions, and changes. Communication between discrepant laboratories and the proficiency test organisers is the best indicator of improvement, since discrepant laboratories usually request advice for managing the discrepancies causes and, later, they inform about their improvements.

Statistic calculations, conclusions, and in the end, reports emitted by participants have also enhanced. The theoretical exercise usefulness relies on illustrate how participants bring their laboratory results to a report that is going to be read by law enforcement agents such as judges and magistrates, attorneys, lawyers, or even other experts. As it has been widely stated, report conclusions are as important as the employed techniques, and GITAD exercise has also done its part to improve them. For instance, some participants changed their conclusions from a categorical statement to a hypothesis contrast.

Finally, collaboration and improvement have also worked together during the 20 years of GITAD. An example of this is the possibility of its members to request courses and workshops about a specific topic, and they are organised during GITAD meetings so they are given by other members with an expertise or demonstrated experience in that field.

### GITAD meetings

GITAD organises a meeting in a different participating country every year: Brazil (Belo Horizonte, 1999; Curitiba, 2001; Brasilia, 2007; Sao Paulo, 2019; Salvador, 2011), Uruguay (Montevideo, 2000), Chile (Santiago, 2002), Mexico (Mexico City, 2005 and 2014; Veracruz, 2013), and Guatemala (Guatemala City, 2010, 2015 and 2018; Online, 2020).

Every GITAD proficiency test exercise involves holding a meeting in one of the participating countries, and those meetings have provided a forum for discussing solutions to the various problems and discrepancies observed, especially transcription errors and lack of resolution, which, as explained earlier, have been the main source of error. Over the last 20 years, the participating laboratories have felt a growing awareness of the quality of their results and have therefore committed to the continuing training of their staff. During GITAD meetings topics related to the work group are treated (assemblies, work commissions), as well as conferences and workshops about topics of interest in forensic genetics.

### Future perspectives

A number of aspects can be considered for improving GITAD proficiency test exercises. First, and most important, is the accreditation under ISO 17043:2010, Conformity assessment-General requirements for proficiency testing, which specifies the general requirements of providers of proficiency test exercises and thus for the operation and development of those proficiency tests, requirements that are supposed to be the basis for particular field applications. Nevertheless, ISO 9001 is already obtained, ISO 17025 is under obtention, and ISO 17043 guidelines for item preparations are undertaken: homogeneity, same matrix, routine items match, sufficient number of items are prepared for participant laboratories and if they are lost or damaged during distribution, and a procedure for acquisition, collection, handling, storage, and disposal of items is already followed by the prepared laboratory. Moreover, most of the items contained in ISO 17043 are met: both technical requirements (personnel, equipment, facilities, program design, planning, method choice, instructions to participants, manipulation and storage of items, packing, labelling, and distribution of items, data analysis and records, performance, reports, communication with participants, and confidentiality) and organisation requirements (almost shared with ISO 17025 requirements of organisation, management system, document control, etc.).

Secondly, some improvements when sending the exercise samples can be made to ensure their reception by the participating laboratories. With that aim, international private courier and package delivery enterprises have been contemplated for next year’s exercise. Lastly, advances in the data collection step could be conducted, elaborating a more homogenous form that could be filled online via a specially designed website.

## Conclusions

Proficiency tests are a fundamental strategy for forensic genetics laboratories in order to assess the accuracy of results, to detect problems, and to implement advancements. Starting with eight participating laboratories, the GITAD proficiency test has now reached 58 participating institutions from Spain and various Latin American countries, as well as Asia, over the last 20 years. Proficiency testing is an important measure for ensuring the quality of results from a forensic genetics laboratory because it helps detect methodological issues that can then be corrected. We have shown the evolution of the various techniques employed for DNA analysis by the participating laboratories, with a clear tendency towards the latest developed techniques and therefore a certain methodology standardisation.

Recognising that effort, several institutions (such as YHRD and the Brazil DNA Database) have accepted the GITAD proficiency test as a valid proficiency test in order to contribute with a DNA profile. However, the strongest indicator of the acceptance of the proficiency test is the growing number of participating laboratories, thanks not only to the exercise’s free-of-charge nature, but also to their aim of being part of a network focused on their action field and the will to achieve high quality results. Furthermore, the degree of trust and demonstrated quality of the providers has an influence in the high ratio of participation.

More than a few lessons have been learned with the exercise over the past 20. Delivering the samples still presents a problem, given that many laboratories cannot receive the items on time, and some do not receive them at all, due to difficulties with customs. A few laboratories have accumulated some of the detected errors, which are connected to the GITAD Strategic Plan 2019–2021 emphasising the need for providing adequate training to the laboratories to implement corrective measures so that errors can be minimised or overcome. For instance, specific topics are addressed during GITAD annual meetings, and if participants ask about a certain one, the laboratories known by their expertise on that matter are invited to give a workshop about it, so members can learn from each other. Finally, a big part of the participant laboratories is accredited under ISO 17025, so the obtention of any discrepancy leads to a formal procedure in order to face the problem. Nevertheless, the best improvement indicator is the direct communication between the participant laboratory and the preparers.

The analysis of the generated data during the exercises gives the proficiency test organisation insight into future perspectives. Four action lines have been established: (1) continuing the growth in the number of participating laboratories, (2) accreditation of the organiser institution under the ISO 17043:2010 to ensure the quality of the proficiency test, (3) improving the delivery of samples to the various laboratories, and (4) developing a website for treating the exercise’s data.

## Supplementary Information

Below is the link to the electronic supplementary material.

Supplementary file1 (PDF 291 KB)
