# Supplementary material for: GITAD 2020: quality assurance test through 20 years of experience
Source: Int J Legal Med. 2022 Feb 22;136(3):659–70. doi: 10.1007/s00414-022-02802-4 (PMC8861261; doi:10.1007/s00414-022-02802-4)

8. Supplementary material

Table 1. Type of forensic sample (M5) since its introduction in 2007

	2007	2008	2009	2010	2011	2012	2013
Sample	1.5 g Fresh bone powder	2 g Fresh bone fragment	35 µl Bloodstain mixture (8:1)	50 µl Semen and saliva stains (1:1)	20 µl Bloodstain mixture (M3 and unknown) (1:2)	15 µl Bloodstain mixture (M3 and unknown) (1:1)	15 µl Bloodstain mixture (M4 and M4 daughter) (1:1)
Support			Filter paper	Filter paper	FTA®	Foam	Foam

	2014	2015	2016	2017	2018	2019	2020
Sample	15 µl Bloodstain mixture (M4 and M4 sister) (1:1)	15 µl Bloodstain mixture (M4 and M4 brother) (1:1)	Touch DNA and 50 µl *Sus scrofa* bloodstain	15 µl Bloodstain mixture (mother and son) (1:1)	Woman hair soaked in M4 blood	15 µl Female and male bloodstain mixtures (1:1, 2:1, 3:1, 5:1)	Cigarette butt with male saliva
Support	FTA®	Filter paper	Filter paper	Filter paper	Filter paper	Human ID Bloodstain Card (Whatman ™)	

Table 2. Type of theoretical exercise and given genetic profiles by year.

	2003	2005	2006	2007	2008	2009
Type of exercise	Paternity	Paternity	Sisterhood	Sisterhood Brotherhood	Sisterhood Brotherhood	Sexual assault
Genetic profiles	Mother Putative son Paternal grandfather Maternal grandmother	Son Putative father	Putative sister 1 Putative sister 2 Putative sister 3	Plaintiff Male 1 Male 2 Plaintiff Female 1 Female 2 Female 3	Plaintiff Male 1 Male 2 Plaintiff Female 1 Female 2 Female 3	Suspect Victim Vaginal swab

	2010	2011	2012	2013	2014	2015
Type of exercise	Paternity	Sisterhood Brotherhood	Uncle-nephew	Half-siblings	Mixture	Paternity (Incest)
Genetic profiles	Mother Son Putative father	Mother 1 Mother 2 Sibling 1 Sibling 2 Putative Sibling 3	M1, M2 (Siblings) M3 (putative nephew)	Mother 1 Plaintiff Son 1 Mother 2 Sibling 1 Sibling 2 Sibling 3	Vaginal swab contributor 1 Vaginal swab contributor 2	Son Putative father

	2016	2017	2018	2019	2020
Type of exercise	Sisterhood	Maternity	Grandparenthood	Disaster Victim Identification	Motherhood
Genetic profiles	Putative sister 1 Putative sister 2	Putative mother Sibling 1 Sibling 2	Putative granddaughter Grandfather Grandmother	NN 1-11 Family 1-11	Putative mother 1 Younger 1-4

	Putative sister 3				Putative mother 2 Younger 1-4

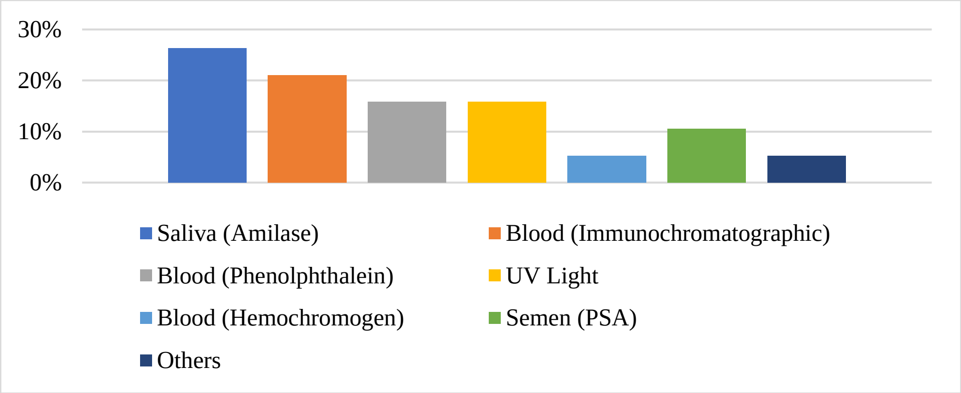

Figure 1. Presumptive tests reported in 2020 GITAD exercise.

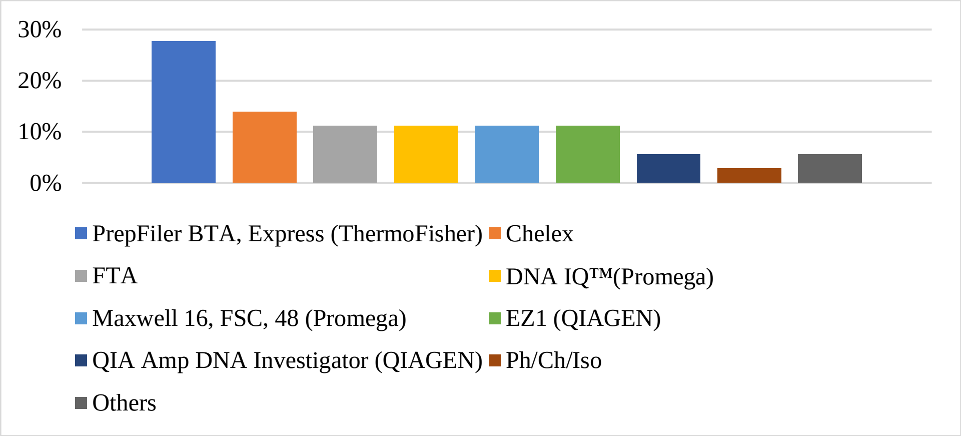

Figure 2. DNA Extraction protocols reported in 2020 GITAD exercise.

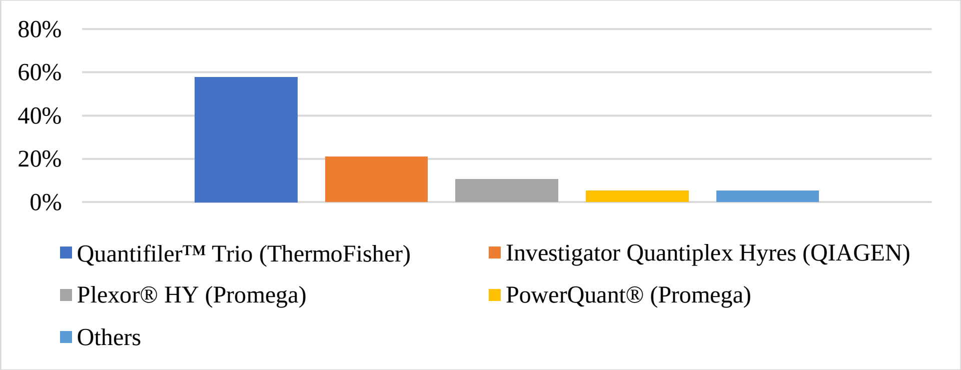

Figure 3. DNA Quantification methods reported in 2020 GITAD exercise.

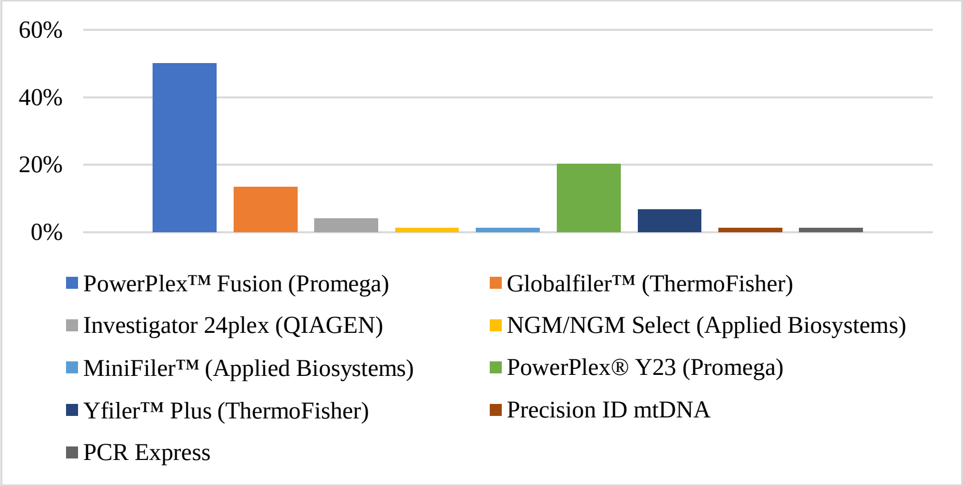

Figure 4. DNA Amplification strategies reported in 2020 GITAD exercise.

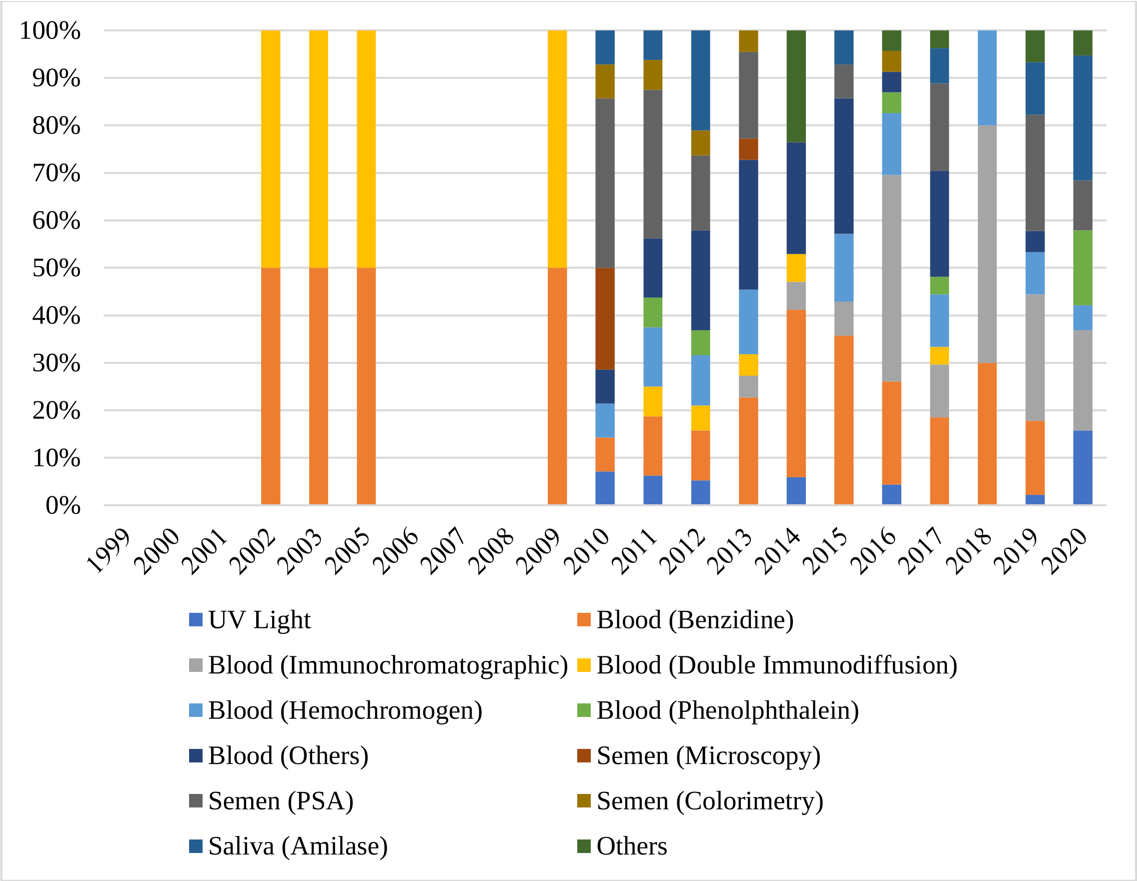

Figure 5. Presumptive tests reported from 1999 to 2020.

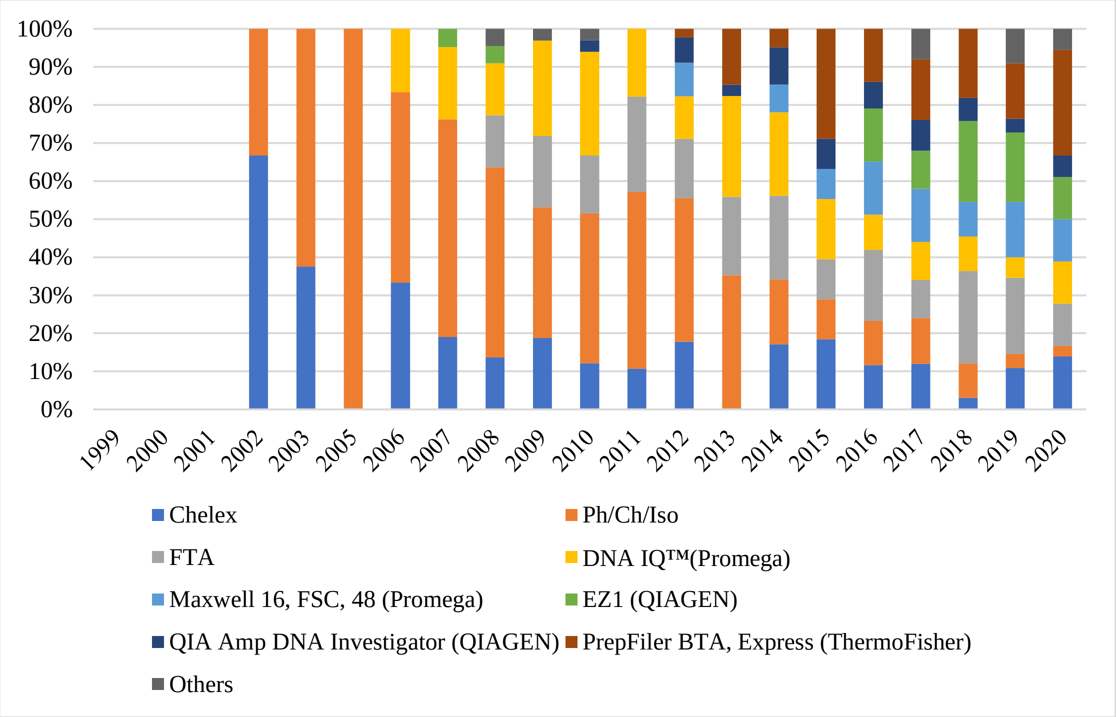

Figure 6. DNA Extraction strategies reported from 1999 to 2020.

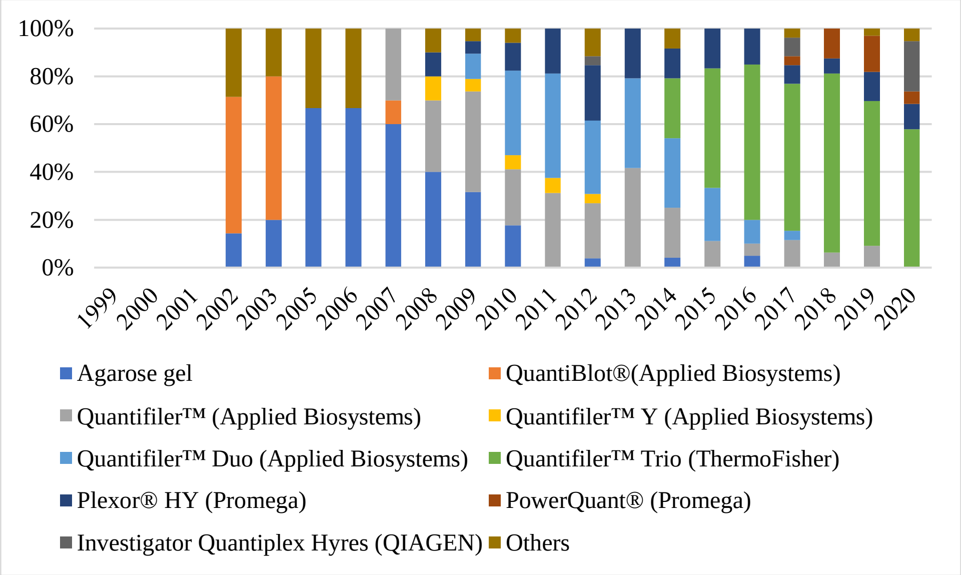

Figure 7. DNA Quantification strategies reported from 1999 to 2020.

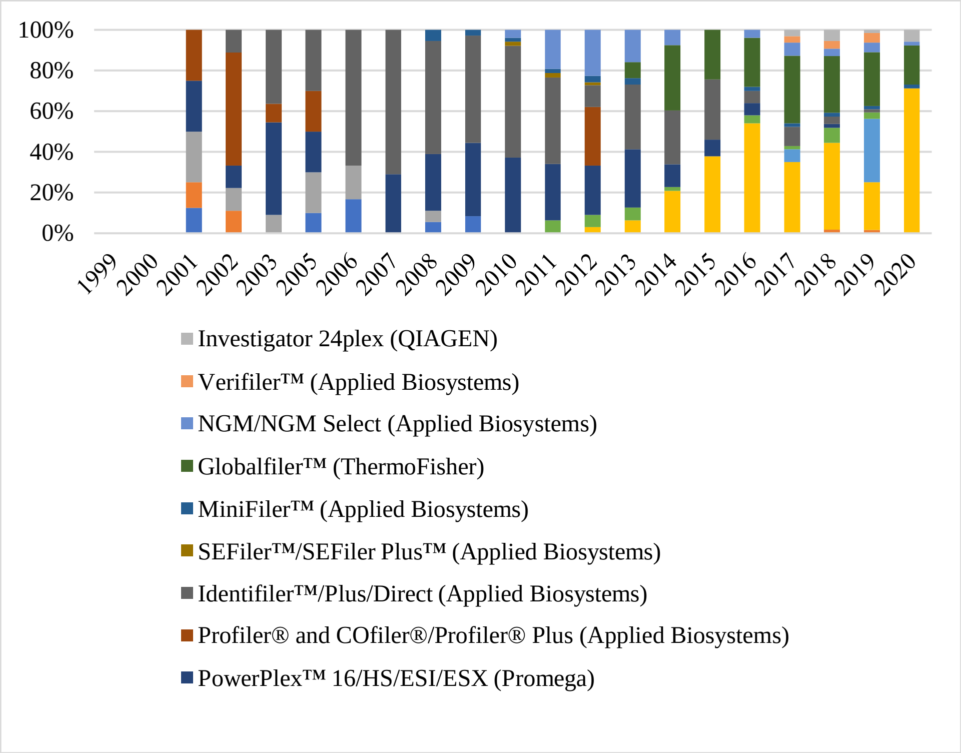

Figure 8. STRs amplification strategies reported from 1999 to 2020.

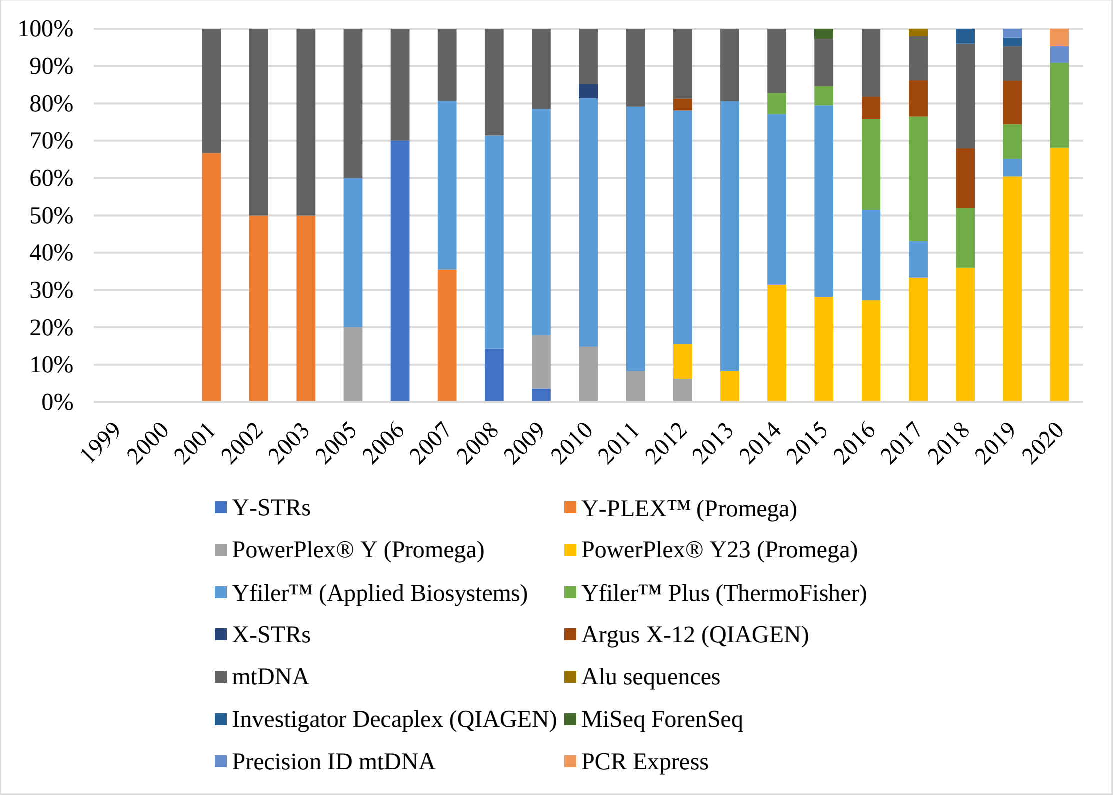

Figure 9. Other amplification strategies reported from 1999 to 2020.

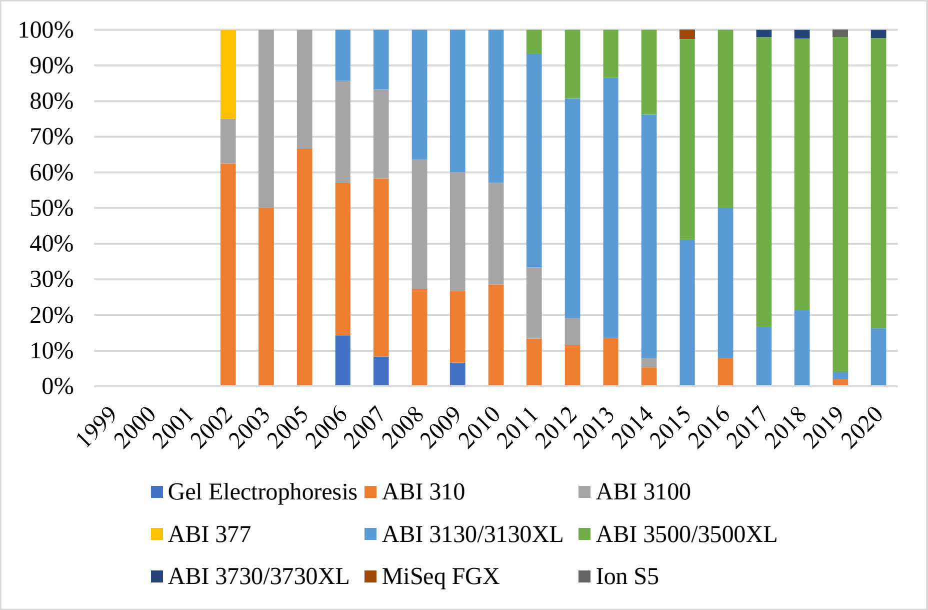

Figure 10. DNA visualization reported from 1999 to 2020.

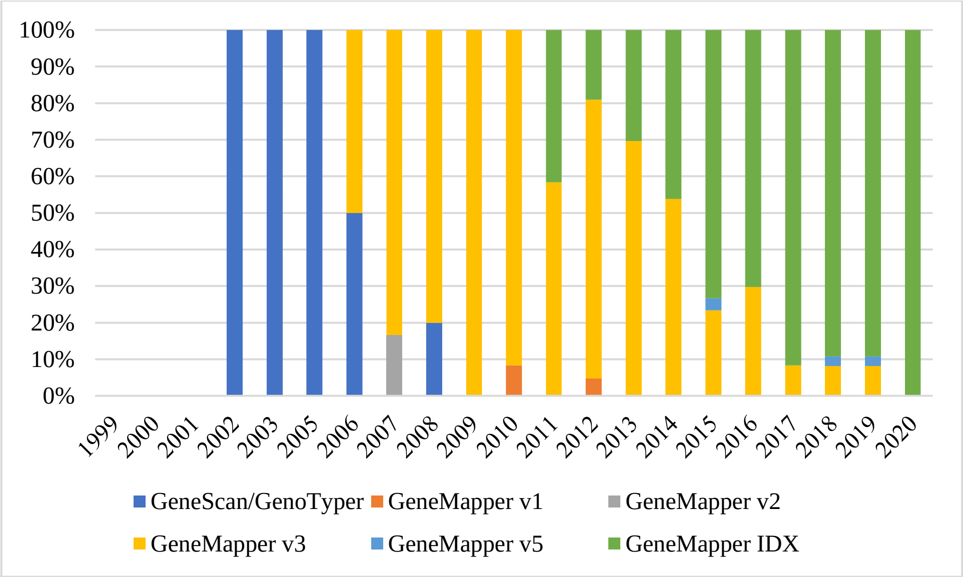

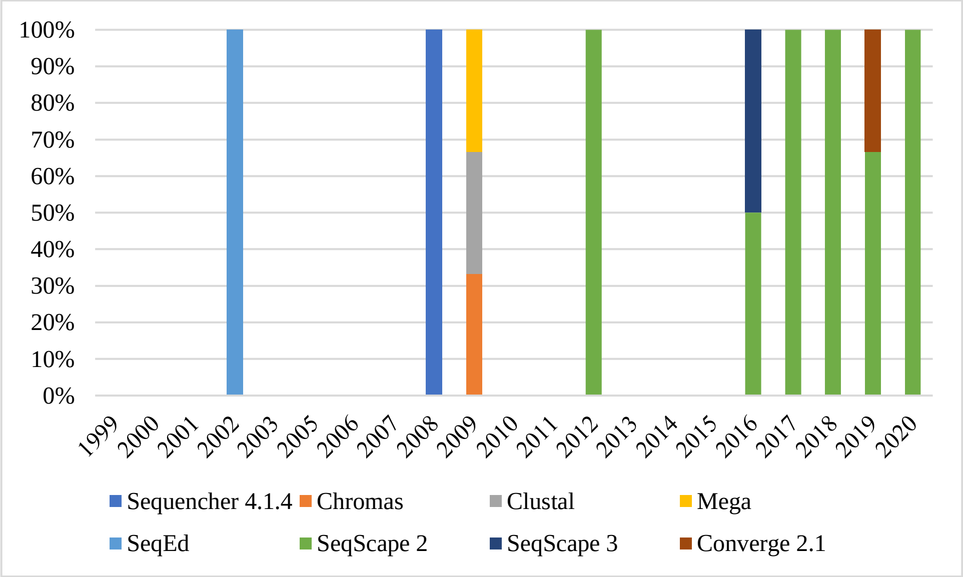

Figure 11. Genotyping software reported from 1999 to 2020.

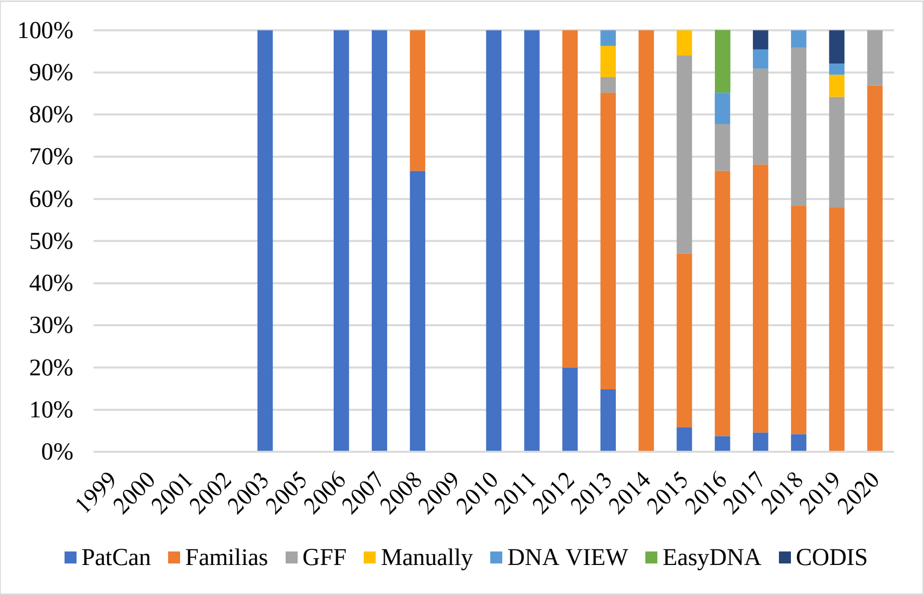

Supplement: Supplementary file 1 — Supplementary file1 (PDF 291 KB) [file 414_2022_2802_MOESM1_ESM.pdf]
